# Lack of Interferon (IFN) Response to T7 Transcribed pppG (n)(n = 2,3)-shRNA

**DOI:** 10.1080/15257770701503647

**Published:** 2007-12-10

**Authors:** Takuma Gondai, Kazuya Yamaguchi, Kahoko Hashimoto, Naoko Miyano-Kurosaki, Hiroshi Takaku

**Affiliations:** Department of Life and Environmental Science, Chiba Institute of Technology, Tsudanuma, Narashino-shi, Chiba, Japan; Department of Life and Environmental Science and High Technology Research Center, Chiba Institute of Technology, Tsudanuma, Narashino-shi, Chiba, Japan

**Keywords:** RNAi, shRNA, T7 RNA polymerase, type I interferon

## Abstract

RNA interference (RNAi) mediated by siRNAs has proved to be a highly effective gene silencing mechanism with great potential for gene therapeutic applications. However, siRNA agents have been shown to exert non-target-related biological effects and toxicities, including immune stimulation. Specifically, siRNA synthesized from a T7 RNA polymerase system can trigger the potent induction of type I IFN in a variety of cells. The single-stranded RNA can also stimulate innate cytokine responses in mammals. We found that pppGn (n = 1–3), associated with the 5′ end of the shRNA produced from the T7 RNA polymerase system, did not induce detectable levels of IFN. The residual amount of G associated with the 5′-end of the transcript was proportional to the reduction of the interferon response. We describe a T7 pppGn (n = 1–3) shRNA synthesis system that alleviates the IFN response, which will facilitate the design of siRNAs while maintaining their full efficacy.

## INTRODUCTION

RNAi is a newly described natural biological phenomenon mediated by siRNA molecules, which target specific mRNAs for degradation by cellular enzymes. Surprisingly, recent studies have indicated that siRNAs can induce global upregulation of the expression of IFN-stimulated genes.^[^1^–^5^]^ This effect was detected with synthetic siRNAs that were transfected into cells and with siRNAs that were produced within cells by the expression of shRNAs. Both of these studies documented significant nonspecific changes in gene expression as a consequence of the delivery of siRNAs. Two recent studies have indicated that the mechanism of the IFN response might include the recognition of the siRNAs by TLR3.^[^4^,^^6^^]^ In addition, long dsRNA and single-stranded (ss) RNA of ssRNA viruses are detected through TLR7 and TLR8,^[^7^,^^8^^]^ located in the endosomal membrane.^[^9^]^ Recently, Kim et al.^[^10^]^ showed that siRNAs synthesized using the T7 RNA polymerase system can trigger the potent induction of IFN-α and -ß in a variety of cells. Themediators of this response revealed that an initiating 5′-triphosphate was required for IFN induction.

In this study, we describe the absence of interferon induction by an new type of shRNAs, pppGG-shRNA, synthesized by bacteriophage polymerase.

## RESULTS AND DISCUSSION

**FIGURE 1:**
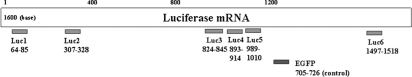
Locations of shRNAs targeting luciferase mRNA.

To investigate the RNAi-mediated silencing of luciferase activity, we initially synthesized six shRNAs targeting the luciferase gene transcript, using T7 RNA polymerase. The locations of these targets are provided in Figure 1. The luc-shRNAs include the 5′-pppGG sequence, because efficient T7 RNA polymerase initiation requires G as the first and second nts of each RNA. The inhibition of luciferase activity was measured by the DLR™ assay system. In the DLR™ assay, the firefly and Renilla luciferase activities are measured sequentially from a single sample. To determine whether pppGGluc-shRNAs can specifically inhibit luciferase gene expression, HeLa CD4^+^ cells were transfected with the pppGG-luc-shRNAs corresponding to the luciferase gene, and then were further transfected with pGL3-control (Firefly) and phRG-TK (Renilla). The six pppGG-luc-shRNAs (luc-1-6) all inhibited the luciferase activities (data not shown), with luc-3 and 4 showing the most inhibitory activity.

**FIGURE 2:**
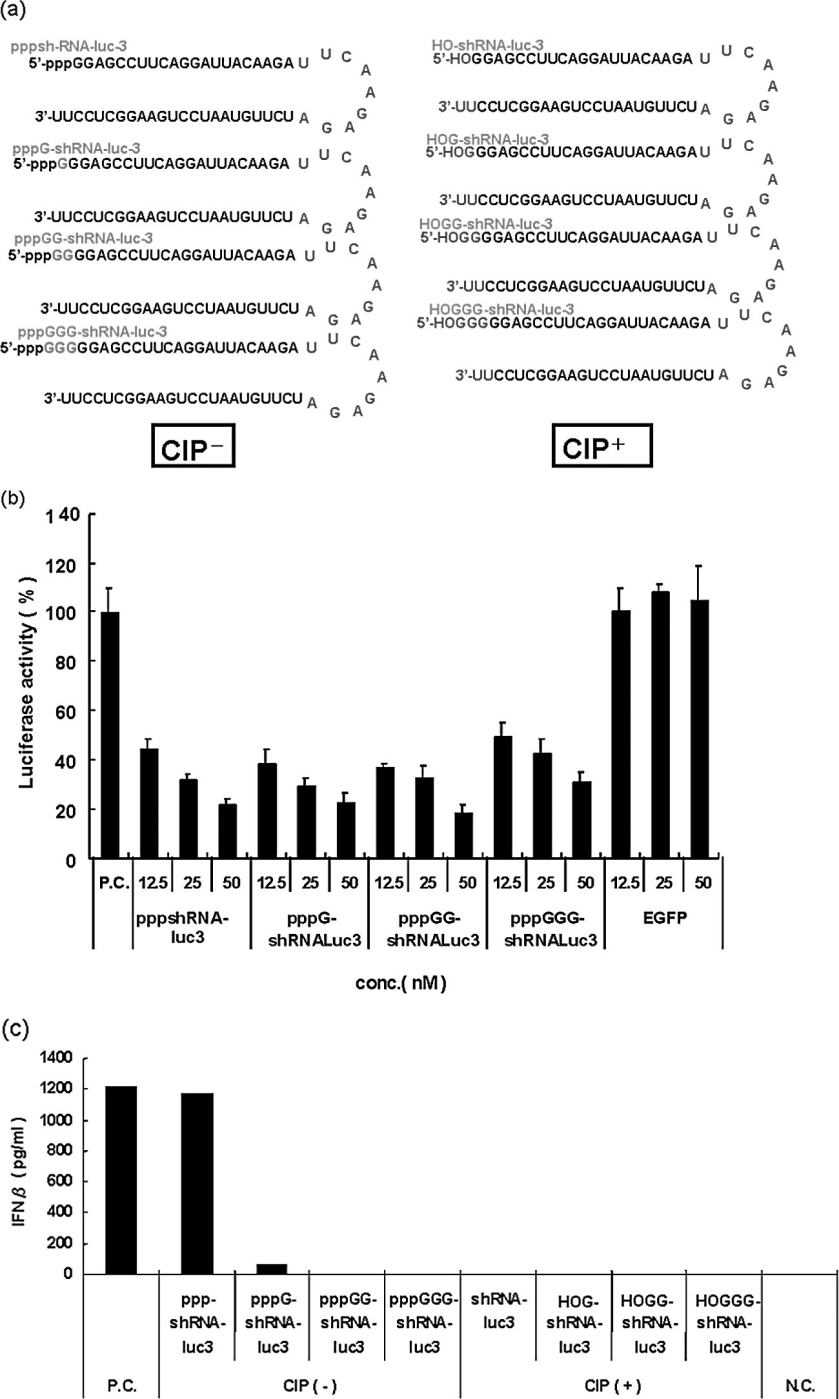
Lack of interferon induction by T7-transcribed shRNA. (a) The shRNA-luc constructs synthesized in these studies. We designed the pppGn (n = 0–3) associated with the 5′ end of the shRNA, which was transcribed by T7 RNA polymerase. We also designed the 5′-HOGn (n = 0–3) with the 5′ end of shRNA (removal of triphosphate by CIP). (b) The anti-luciferase activity of the pppGn (n = 0–3) associated with the 5′ end of shRNA-luc-3 in HeLaCD4^+^ cells. Firefly and Renilla luciferase activities were measured consecutively by using dual-luciferase assays (Promega) 48 h after transfection. (c) The residual amount of G associated with the 5′-end of the transcript is essential for the lack of interferon induction. HeLaCD4^+^ cells were transfected with 100 nM of pppGn (n = 0–3)-shRNAs or HOGn (n = 0–3)-shRNAs. The induced levels of IFN-β were determined by an ELISA.

Recently, Kim et al.^[^10^]^ showed that siRNAs synthesized using the T7 RNA polymerase system can trigger the potent induction of IFN-α and -ß in a variety of cells. The mediators of this response revealed that an initiating 5′-triphosphate was required for IFN induction. To verify the induction of INF by pppGG-shRNA, we designed pppGn (n = 0–3) associated with the 5′ end of shRNA-luc-3, which was transcribed by T7 RNA polymerase (Figure 2a). The luc-3 construct induced the inhibition of luciferase activity in a dose-responsive manner (Figure 2b). However, the control pppGGshRNA, targeting EGFP itself, showed no inhibitory effect on the luciferase activity (Figure 2b). Furthermore, the residual amount of G associated with the 5′-end of the transcript did not affect the inhibition of the luciferase activity. On the other hand, we also designed the 5′-HOGn (n = 0–3) at the 5′ end of the shRNA and tested these shRNA for IFN induction (Figure 2a). We assayed the media from pppGn or 5′-HOGn (n = 0–3)-shRNA transfected HeLa CD4^+^ cells for IFN-ß, using an enzyme-linked immunosorbent assay (ELISA). Interferon assays from the pppGn (n = 2,3) associated with 5′ end of shRNA revealed no interferon induction in HeLa CD4^+^cells (Figure 2c). The interferon response was initiated slightly by the pppGn (n = 1) associated with the 5′ end of the shRNA. The pppGn (n = 0) associated with the 5′ end of the shRNA showed more potent IFN induction than the pppGn (n = 1) associated with the 5′ end of the shRNA. However, no IFN induction in HeLa CD4^+^ cells was elicited by the 5′-HOGn (n = 1–3) (Figure 2c). Furthermore, the inhibition of luciferase activity by pppGn(n = 0)-luc-shRNA observed in our data (Figure 2b–c) could be partly due to IFN induction. These results suggest that the residual amount of G associated with the 5′-end of the transcript was proportional to the reduction of the IFN response.

## Abbreviations

siRNA: small interfering RNA
shRNA: short hairpin RNA
TLR: Toll-like receptor
luc: luciferase
DLR™: dual-luciferase reporter
HeLa CD4^+^ cells: HeLa cells transfected with human CD4.
